# New Mutations in DNHD1 Cause Multiple Morphological Abnormalities of the Sperm Flagella

**DOI:** 10.3390/ijms24032559

**Published:** 2023-01-29

**Authors:** Guillaume Martinez, Anne-Laure Barbotin, Caroline Cazin, Zeina Wehbe, Angèle Boursier, Amir Amiri-Yekta, Abbas Daneshipour, Seyedeh-Hanieh Hosseini, Nathalie Rives, Aurélie Feraille, Nicolas Thierry-Mieg, Marie Bidart, Véronique Satre, Christophe Arnoult, Pierre F. Ray, Zine-Eddine Kherraf, Charles Coutton

**Affiliations:** 1 CHU Grenoble Alpes, UM de Génétique Chromosomique, 38000 Grenoble, France; 2 Team Genetics Epigenetics and Therapies of Infertility, Institute for Advanced Biosciences, University Grenoble Alpes, INSERM U1209, CNRS UMR 5309, 38000 Grenoble, France; 3 CHU Lille, Institut de Biologie de la Reproduction-Spermiologie-CECOS, 59000 Lille, France; 4 CHU de Grenoble, UM GI-DPI, 38000 Grenoble, France; 5 Department of Genetics, Reproductive Biomedicine Research Center, Royan Institute for Reproductive Biomedicine, ACECR, Tehran 16635-148, Iran; 6 Department of Andrology, Reproductive Biomedicine Research Center, Royan Institute for Reproductive Biomedicine, ACECR, Tehran 16635-148, Iran; 7 Team Adrenal and Gonadal Pathophysiology, Inserm, U1239 NorDIC, University Rouen Normandie, 76000 Rouen, France; 8 Reproductive Biology Laboratory—CECOS, Rouen University Hospital, 76000 Rouen, France; 9 TIMC, University Grenoble Alpes, CNRS, UMR 5525, 38000 Grenoble, France; 10 CHU Grenoble Alpes, Laboratoire de Génétique Moléculaire: Maladies Héréditaires et Oncologie, 38000 Grenoble, France

**Keywords:** MMAF, *DNHD1*, teratozoospermia, genetics of male infertility, whole-exome sequencing

## Abstract

Male infertility is a common and complex disease and presents as a wide range of heterogeneous phenotypes. Multiple morphological abnormalities of the sperm flagellum (MMAF) phenotype is a peculiar condition of extreme morphological sperm defects characterized by a mosaic of sperm flagellum defects to a total asthenozoospermia. At this time, about 40 genes were associated with the MMAF phenotype. However, mutation prevalence for most genes remains individually low and about half of individuals remain without diagnosis, encouraging us to pursue the effort to identify new mutations and genes. In the present study, an a cohort of 167 MMAF patients was analyzed using whole-exome sequencing, and we identified three unrelated patients with new pathogenic mutations in *DNHD1*, a new gene recently associated with MMAF. Immunofluorescence experiments showed that DNHD1 was totally absent from sperm cells from *DNHD1* patients, supporting the deleterious effect of the identified mutations. Transmission electron microscopy reveals severe flagellum abnormalities of sperm cells from one mutated patient, which appeared completely disorganized with the absence of the central pair and midpiece defects with a shortened and misshapen mitochondrial sheath. Immunostaining of IFT20 was not altered in mutated patients, suggesting that IFT may be not affected by *DNHD1* mutations. Our data confirmed the importance of DNHD1 for the function and structural integrity of the sperm flagellum. Overall, this study definitively consolidated its involvement in MMAF phenotype on a second independent cohort and enriched the mutational spectrum of the *DNHD1* gene.

## 1. Introduction

Infertility is one of the most alarming public health issues supported by contemporary evidences demonstrating a constant and important decline in global fertility and in sperm parameters during the past decades [1]. Male factors are involved in up to half of all infertile couples, and about 7% of the male population may be concerned [2]. Male infertility is a common and complex disease and presents as a wide range of heterogeneous phenotypes, ranging from “apparently” normal semen parameters to the complete absence of sperm production, passing through one or a combination of low sperm concentration, poor sperm motility, or abnormal morphology. Although often multifactorial, it is accepted that male infertility has an important genetic component with a prevalence all the higher as the forms are severe [3]. Identifying the genes causing male infertility is challenging and important to increase the understanding of the physiopathological processes involved in this condition as well as the diagnostic yield [4]. Significant advances have been made, in particular, for severe qualitative sperm defects, as shown by the prolific identification of numerous genes in the multiple morphological abnormalities of the sperm flagellum (MMAF) phenotype, which is a condition of extreme morphological sperm defects characterized by a mosaic of sperm cells with short, coiled, absent, or irregular flagellum associated with severe asthenozoospermia [5]. Severe disorganization of axonemal and periaxonemal structures is observed at the ultrastructural level, and loss of the central pair, peripheral microtubule doublets, or dynein arms are common.

At this time, about 40 genes are associated with MMAF phenotype [6]. Several gene families are strongly involved in the MMAF phenotype, such as the genes encoding dynein axonemal heavy chain (*DNAH1*, *DNAH2*, *DNAH6*, *DNAH17*), cilia and flagella associated proteins (*CFAP43*, *CFAP44*, *CFAP65*, *CFAP69*, *CFAP70*, *CFAP75*, *CFAP251*), and tetratricopetide repeat proteins (*TTC21A*, *TTC29*). Several other genes outside the main families have also been reported: encoding fibrous sheath interaction protein (*FSIP2*), sperm flagellum protein 2 (*SPEF2*), centrosomal protein (*CEP135*), or armadillo repeat-containing protein (*ARMC2*), among others (see [6] for review). However, mutation prevalence for most genes remains individually low, and about half of individuals remain without diagnosis, demonstrating the high genetic heterogeneity of this phenotype. The knowledge of the genetic architecture of the MMAF phenotype allowed us to improve our knowledge about the physiopathological mechanisms involved in the sperm phenotype and to optimize the medical care and management of infertile male. Overall, this encourages us to pursue the effort to identify new mutations and/or new genes in this phenotype.

Whole-exome sequencing (WES) has proven to be a valuable tool for identifying potential genetic contributors to male infertility, including multiple morphological abnormalities of sperm flagella [7,8,9,10,11,12,13,14,15,16,17,18,19,20,21,22,23,24,25,26,27]. This technique allows rapid and cost-effective analysis of a large number of genes at once, making it a useful tool for studying complex genetic conditions such as infertility, although it does not allow visualization of noncoding regions. In a cohort of 167 MMAF patients, 83 patients with harmful mutations in known MMAF-related genes were previously identified using WES [7,8,9,10,11,12,13,14,15,16,17,18,19,20,21,22,23,24,25,26,27], leading to a 49.7% diagnostic rate. In the present study, a reanalysis of this cohort using whole-exome sequencing allowed identification of novel homozygous mutations in *DNHD1* (dynein heavy chain domain 1), also known as *CCDC35* (coiled-coil domain-containing protein 35), a 14 876 base pair gene located in the p15.4 region of chromosome 11. It codes for the 4753 amino acid protein dynein heavy chain domain 1, containing five coiled-coil domains and predominantly expressed in the sperm flagellum. Similar to the gene groups mentioned above, more than twenty coiled-coil domain-containing proteins are known to be associated to male infertility [28], including *DNHD1*, as Tan et al. recently identified bi-allelic deleterious *DNHD1* variants in eight unrelated men with MMAF phenotype [29]. They thus reported two homozygous (one stop-gain and one missense mutations) and six compound heterozygous mutations (five missense, four stop-gain and three frameshift mutations) that all lead to an MMAF phenotype with asthenozoospermia. They also generated *Dnhd1*^−/−^ mice that showed similar defects and further confirmed the involvement of *DNHD1* in the phenotype.

The achieved aim of this study was to definitively consolidate *DNHD1* involvement in MMAF phenotype on a second independent cohort, enrich the mutational spectrum of the *DNHD1* gene, and confirm its importance for the function and structural integrity of the sperm flagellum.

## 2. Results

### 2.1. Whole-Exome Sequencing (WES) Identifies Pathogenic Mutations in DNHD1 in MMAF Individuals

**Figure 1:**
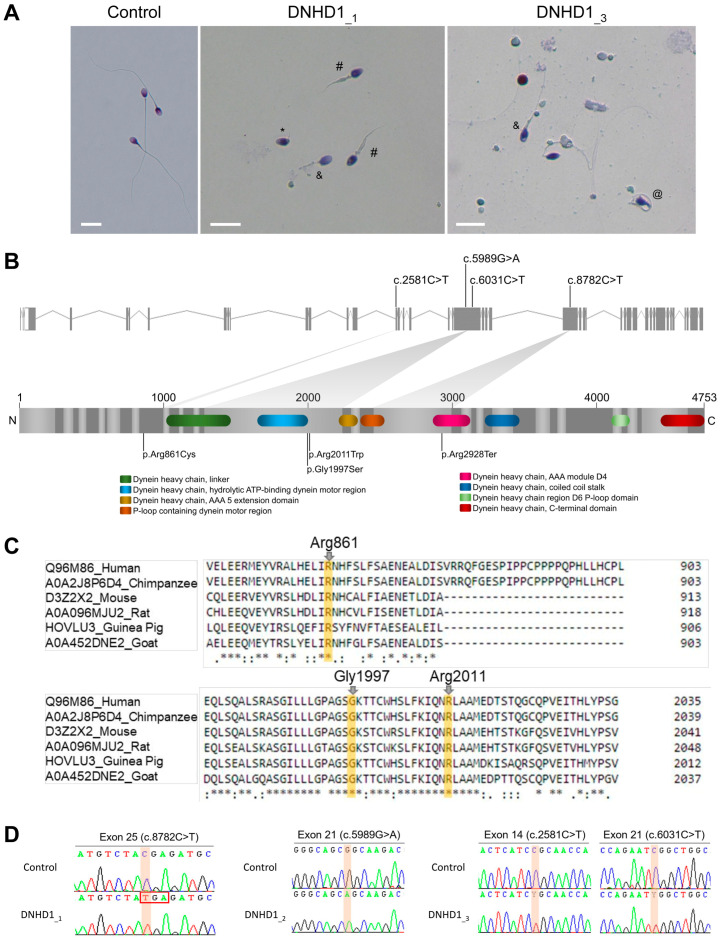
Sperm morphology and description of the identified *DNHD1* variants. (**A**) Light microscopy analysis of spermatozoa from fertile control individual, patient DNHD1__1_ and patient DNHD1__3_. Most spermatozoa from *DNHD1* patients have flagella that are short (#), absent (*), coiled (@) or of irregular caliber (&). Head malformations were also observed. Scale bar = 10 µm. (**B**) *DNHD1* gene structure (ENST00000254579.11) showing the location of the variants described. The functional structure of the encoded protein is shown in the lower panel. Colored boxes on the protein represent functional domains according to UniProtKB (Q96M86) and the DECIPHER database. (**C**) Conservation and alignment of DNHD1 sequences from several orthologs around the missense mutated amino acids reported in the present study (marked by a yellow square). (**D**) Electropherograms obtained by Sanger sequencing showing the homozygous mutations c.8782C>T and c.5989G>A identified in the patients DNHD1__1_ and DNHD1__2_, respectively, and the two heterozygous variants (c.2581C>T and c.6031C>T) of *DNHD1* identified in subject DNHD1__3_.

Reanalysis of the remaining exomes of a 167 MMAF patients cohort allowed identification of three additional unrelated patients (DNHD1__1–3_) with MMAF phenotype (Figure 1A, Table 1). One originated from Iran (DNHD1__2_) and two from Europe (DNHD1__1and3_), and harbored pathogenic variants in *DNHD1*, accounting for 1.8% of our cohort. For all individuals, no variants with low frequency in control databases were identified in other genes reported to be associated with cilia, flagella, or male fertility.

The patient DNHD1__1_ carried a stop-gain variant c.8782C>T; p.(Arg2928Ter) (NM_144666.3) located in the exon 25 (Figure 1B). The variant is present in the Genome Aggregation Database (gnomAD v2.1, http://gnomad.broadinstitute.org/, accessed on 6 September 2022) with a minor allele frequency (MAF) of 1.14 × 10^−3^ and was previously reported as pathogenic [29]. The two other patients (DNHD1__2and3_) harbored missense variants. The patient DNHD1__2_ had a homozygous missense variant c.5989G>A; p.(Gly1997Ser) located in the exon 21 (Figure 1B). The patient DNHD1__3_ was identified to be compound heterozygous c.2581C>T; p.(Arg861Cys) and c.6031C>T; p.(Arg2011Trp). Unfortunately, pedigree analysis could not be performed for this patient to confirm the transmission of the variants because the samples of his parents were not available. All these missense variants affect conserved residues among species (Figure 1C), are found at a low prevalence in the general population according to the GnomAD database, and are predicted to be likely damaging by most of the in silico bioinformatics tools used (Table S1). This support the hypothesis that the identified missense *DNHD1* variants may be responsible for the observed infertility phenotypes. All mutations identified by exome sequencing were validated by Sanger sequencing (Figure 1D, Table S2).

**Figure 2:**
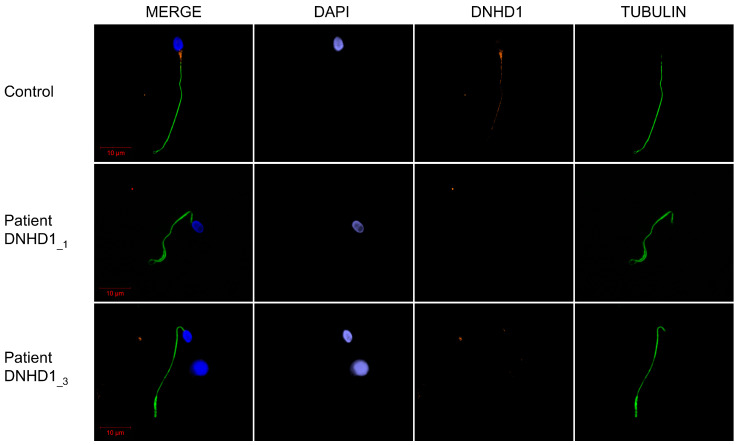
DNHD1 immunostaining of sperm cells from patients with *DNHD1* mutation. Sperm cells from a fertile control individual and the two DNHD1__1_ and DNHD1__3_ patients stained with anti-DNHD1 (orange) and anti-acetylated tubulin (green) antibodies. DNA was counterstained with DAPI (blue). In the fertile control, DNHD1 was present throughout the entire flagella with a higher intensity in the midpiece of the sperm flagellum. Conversely, DNHD1 was totally absent from all sperm cells from both individuals DNHD1__1_ and DNHD1__3_. Scale bars: 10 µm.

To further investigate the pathogenicity of the *DNHD1* variants identified, the distribution of DNHD1 in sperm cells from control and *DNHD1* patients was investigated by immunofluorescence staining. Due to sample availability, these analyses were only carried out for individuals DNHD1__1_ and DNHD1__3_. In sperm from control individuals, the DNHD1 staining was present throughout the entire flagella with a high intensity in the midpiece of the sperm flagellum (Figure 2). Conversely, DNHD1 was totally absent from all sperm cells from both individuals DNHD1__1_ and DNHD1__3_ (Figure 2), demonstrating a deleterious effect of the respective *DNHD1* mutations on the protein stability. In order to determine if the loss of DNHD1 protein was a constant feature of the MMAF phenotype, irrespective of the genotype, three MMAF patients carrying mutations in ARMC2, WDR66, FSIP2, and one MMAF patient with unknown genetic cause identified were analyzed. In all these patients, DNHD1 immunostaining was comparable with that observed in fertile controls (Figure S1), suggesting that the absence of DNHD1 observed in DNHD1__1_ and DNHD1__3_ is likely due to the *DNHD1* mutation identified.

Compared to sperm from control samples, sperm from each individual showed severe defects characteristic of MMAF (Figure 1A, Table 1). Semen parameters of individuals carrying *DNHD1* mutations were compared. Very low sperm concentrations and total sperm counts in the ejaculates from the two patients DNHD1__1_ and DNHD1__3_ (Table 1) were observed. The progressive motility rate in DNHD1__2_ decreased dramatically to zero, while the DNHD1__1_ and DNHD__3_ had progressive motility rates of between 10% and 3.5%, respectively (Table 1). A high rate of head malformations, in particular acrosome defects, was also observed in DNHD1__1_ and DNHD__3_ (Table 1).

### 2.2. DNHD1 Variants Lead to a Severe Axonemal Disorganization of the Sperm Flagellum but Does Not Seem to Affect the Intra-Flagellar Transport (IFT)

**Figure 3:**
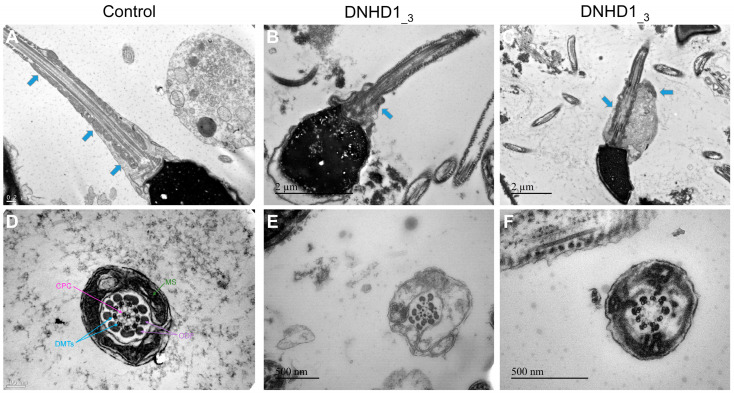
Transmission electron microscopy analyses of sperm cells from a control individual (**A**,**D**) and the patient DNHD1__3_ (**B**,**C**,**E**,**F**). In longitudinal sections (**B**,**C**), a short tail can be noticed corresponding to a cytoplasmic mass containing the different components of the flagellum, all disorganized. In addition, severe midpiece defects are present with a shortened and misshapen mitochondrial sheath. In cross-sections of sperm flagellum from the patients (**E**,**F**), some sections displayed a 9 + 0 axoneme lacking the central pair complex with an abnormal number of outer dense fibers. CPC: central pair complex; DMTs/peripheral doublet microtubules; MS: mitochondrial sheath; ODF: outer dense fibers. Blue arrows mark the mitochrondrial sheath in longitudinal sections.

Transmission electron microscopy (TEM) was used to investigate the ultrastructure of sperm cells from the mutated patient DNHD1__3_ carrying the two missense variants, c.2581C>T; p.(Arg861Cys) and c.6031C>T; p.(Arg2011Trp), leading to the absence of the DNHD1 evidenced previously by IF (Figure 3). Due to the low number of available sperm cells, only a few cross-sections (<10) presented a sufficient quality to be analyzed, and, therefore, no statistical analysis could be conducted. Among these cross-sections, abnormal conformations were observed with, in particular, the absence of the central pair complex (CPC) (9 + 0 conformation) (Figure 3F). In some sections, peri-axonemal structural defects were observed, such as an abnormal number of outer dense fibers (Figure 3E), a defect already observed in sperm from MMAF-affected patients carrying variants in other genes. Observation of longitudinal sections showed severe flagellum abnormalities, which appeared completely disorganized, resulting in truncated flagella or a cytoplasmic mass encompassing unassembled axonemal components (Figure 3C). In addition, severe midpiece defects were present with poorly assembled and misshapen mitochondrial sheaths (Figure 3B).

**Figure 4:**
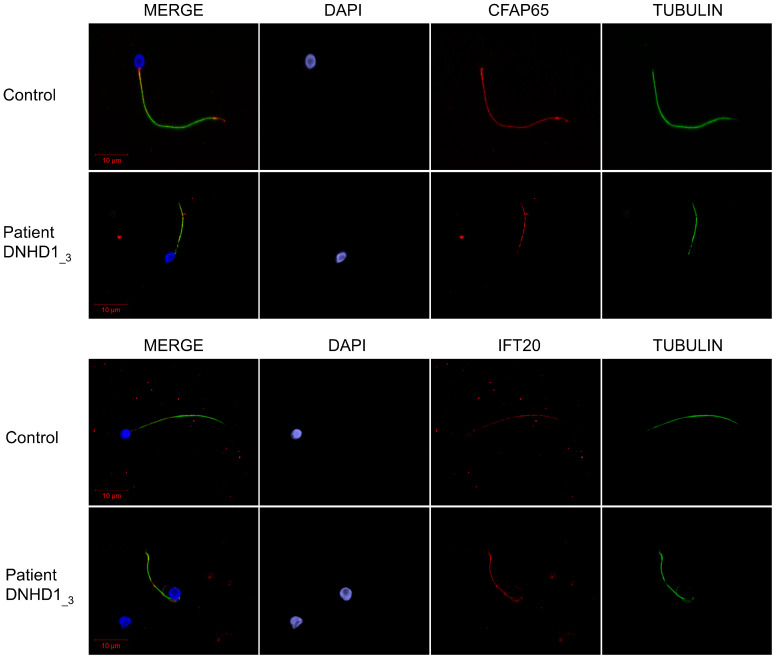
CFAP65 and IFT20 immunostainings of sperm cells from DNHD1 patients. Sperm cells from a fertile control and DNHD1_3 stained with anti-CFAP65 (red) and anti-acetylated tubulin (green) antibodies. DNA was counterstained with DAPI (blue). CFAP65 immunostaining is present throughout the flagellum with no differences between control and patient. Sperm cells from a fertile control and DNHD1__3_ stained with anti-IFT20 (red) and anti-acetylated tubulin (green) antibodies. DNA was counterstained with DAPI (blue). Immunostaining for IFT20 was comparable with control, suggesting that IFT may be not directly affected by mutations in DNHD1. Scale bars: 10 µm.

Mitochondrial sheath malformation is an atypical defect observed in MMAF patients and was reported for few genes such as *CFAP65* [30]. It has also been previously suggested that the absence of DNHD1 may disrupt the interaction with proteins involved in the intra-flagellar transport (IFT) process or affect the expression of IFT-related proteins leading to the abnormal flagellar assembly and, in particular, to the peri-axonemal defects [29]. To explore this assumption and to evidence a possible interaction between DNHD1 and CFAP65, subsequent study of the presence and localization of the IFT-related proteins IFT20 and CFAP65 were investigated. However, immunostaining for IFT20 and CFAP65 were similar to controls, indicating that IFT does not seem to be affected by *DNHD1* mutations and that CFAP65 does not directly interact with DNHD1 into the same axonemal complex (Figure 4).

## 3. Discussion

The present work reports new pathogenic *DNHD1* variants in MMAF patients, thus confirming that this gene is necessary for sperm flagellum structure and function. When adding the three *DNHD1* mutated subjects of the cohort, a diagnostic efficiency of 51.4% (86/167) is obtained, demonstrating the efficiency and the clinical utility of WES to investigate the genetic causes of MMAF syndrome. However, despite regular new gene or variant identification, about half of MMAF individuals remain with unknown genetic causes, highlighting the high genetic heterogeneity of the phenotype consistent with the large number of genes involved in spermatogenesis [31]. These results also suggest that the WES approach cannot be expected to provide 100% positive diagnoses. This may be explained in part by the fact that some variants are not detected by the technique used (deep intronic variants, insufficient coverage) or by the current bio-informatic pipeline used for the analysis (e.g., small duplications, structural variants rearrangements). To improve this diagnosis rate, more powerful techniques such as whole genome sequencing (WGS) may now be envisaged for MMAF patients with WES negative results [32]. 

One of the main difficulties associated with WES is the confirmation of the deleterious effect of the identified variant. The effect of the first *DNHD1* variant c.8782C>T; p.(Arg2928Ter) cannot really be questioned as it led to a stop gain. The effects of the three other missense variants are more complicated to predict. However, several arguments are in favor of their pathogenicity: (i) the three variants were described as deleterious by most prediction tools; (ii) their low prevalence in the general population; and (iii) they affect a conserved residue location (Figure 1C, Table S1). In addition, the DNHD1 protein was shown to be absent from the mutated patient (DNHD1__3_) carrying compound heterozygous c.2581C>T; p.(Arg861Cys) and c.6031C>T; p.(Arg2011Trp) (Figure 2). Unfortunately, IF experiments could not be carried out in DNDH1__2_ due to a lack of sperm cells available. However, the homozygous missense variant p.(Gly1997Ser) is located in the hydrolytic ATP binding site of the protein, reinforcing the potential pathogenic effect of the mutation (Figure 1). Interestingly, pathogenic missense mutations in *DNHD1* were regularly reported in MMAF patients [29] and seem to be a recurrent disease mechanism. Several of these missense mutations are concentrated in the exon 21 [29] (Figure 1). Although the exon 21 is the largest exon of the *DNDH1* gene, this observation could suggest that the exon 21 is critical for the function of DNHD1 and may be a mutational hotspot. Conversely to what was observed for other MMAF genes such as *DNAH1* [15], this study did not observe a clear genotype–phenotype correlation depending on the severity of *DNHD1* mutations (Table 1).

Several ultrastructural axonemal and peri-axonemal components of the flagellum were found to be affected by the absence of the DNHD1 protein. First, CPC anomalies were shown by TEM experiments (Figure 3), highlighting that the CPC defect is a recurrent feature of the MMAF phenotype irrespective to the genotype [33]. More specifically, mitochondrial sheath malformations were observed (Figure 3), which is an atypical defect observed in MMAF patients only reported for few genes such as CFAP65 [31]. It was suggested that such ultrastructural defects might be associated with IFT defects [29]. However, the persistence of IFT20 and CFAP65 in sperm cells from *DNHD1* patients tended to demonstrate that IFT does not seem to be affected by *DNHD1* mutations and that CFAP65 does not directly interact with DNHD1 into the same structural or functional complex. Additional experiments should now be performed to formally conclude about the role of DNHD1 in the flagellar assembly.

Altogether, our data confirmed the importance of DNHD1 for the function and structural integrity of the sperm flagellum. This study definitively consolidated its involvement in MMAF phenotype and enriched the mutational spectrum of the *DNHD1* gene.

## 4. Materials and Methods

### 4.1. Human Subjects and Controls

With the aim of identifying further genetic causes associated with flagellum malformations, we analyzed whole-exome sequencing data from a cohort of 167 MMAF individuals previously established by our team [8]. All patients presented with a typical MMAF phenotype characterized by severe asthenozoospermia (total sperm progressive motility below 10%) with at least three of the following flagellar abnormalities present in >5% of the spermatozoa: short, absent, coiled, bent, or irregular flagella [8]. All patients had a normal somatic karyotype (46, XY) with normal bilateral testicular size and normal hormone levels and secondary sexual characteristics. Sperm analysis was carried out in the source laboratories during routine biological examination of the patients according to World Health Organization (WHO) guidelines [34]. The morphology of the patients’ sperm was assessed with Papanicolaou staining. Detailed semen parameters of the three *DNHD1* mutated patients (DNHD1__1–3_) are presented in Table 1. Sperm samples for additional phenotypic characterization could only be obtained from DNHD1__1_ and DNHD1__3_. Informed consents were obtained from all the patients participating in the study according to local protocols and the principles of the Declaration of Helsinki. The study was approved by local ethics committees, and samples were then stored in the Fertithèque collection declared to the French Ministry of health (DC-2015-2580) and the French Data Protection Authority (DR-2016-392).

### 4.2. Sanger Sequencing

*DNHD1* variants identified by exome sequencing were validated by Sanger sequencing as previously described [8]. PCR primers and protocols used for each patient are listed in Table S2.

### 4.3. Immunostaining in Human Sperm Cells

Immunofluorescence (IF) experiments were performed using sperm cells from fertile control individuals, the patient DNHD1__1_ carrying the homozygous stop-gain variant c.8782C>T; p(Arg2928Ter), the patient DNHD1__3_ carrying the two missense variants c.2581C>T; p.(Arg861Cys) and c.6031C>T; p.(Arg2011Trp), and four another MMAF patients, including three patients with pathogenic mutations in *ARMC2*, *WDR66*, *FISP2*, previously described [8,17,22], and one with unknown genetic cause identified. For each studied MMAF patient, 200 sperm cells were analyzed by two different experienced operators and the IF staining intensity and pattern were compared with a fertile control. Sperm cells were fixed in phosphate-buffered saline (PBS)/4% paraformaldehyde for 1 min at room temperature. After washing in 1 mL PBS, the sperm suspension was spotted onto 0.1% poly L-lysine pre-coated slides (Thermo Fisher Scientific, Waltham, MA, USA). After attachment, sperm were permeabilized with 0.1% (*v*/*v*) Triton X-100-DPBS (Triton X-100; Sigma-Aldrich Co., Ltd., Irvine, UK) for 5 min at RT. Slides were then blocked in 5% normal serum–DPBS (normal goat or donkey serum; GIBCO, Thermo Fisher Scientific) and incubated overnight at 4 °C with the primary antibodies: rabbit polyclonal anti-DNHD1 (PAB15797, Abnova (Taipei, Taiwan), 1:100), rabbit polyclonal anti-IFT20 (PA5-54394,Life Technologies, 1:100), rabbit polyclonal anti-CFAP65 (HPA063406, Sigma-Aldrich, 1:100), and monoclonal mouse anti-acetylated-α-tubulin (AB61601, Abcam (Cambridge, UK), 1:400). Washes were performed with 0.1% (*v*/*v*) Tween 20–DPBS, followed by 1 h incubation at room temperature with secondary antibodies. Highly cross-adsorbed secondary antibodies (Dylight 488 and Dylight 549, 1:1000) were from Jackson Immunoresearch^®^ (Cambridgeshire, UK). Appropriate controls were performed, omitting the primary antibodies. Samples were counterstained with 5 mg/mL Hoechst 33342 (Sigma-Aldrich) and mounted with DAKO mounting media (Thermo Fisher Scientific). Fluorescence images were captured with a confocal microscope (Zeiss LSM 710).

### 4.4. Transmission Electron Microscopy

Transmission Electron Microscopy (TEM) experiments were performed using sperm cells from control individuals and from DNHD1__3_ carrying the two missense mutations c.2581C>T; p.(Arg861Cys) and c.6031C>T; p.(Arg2011Trp). Following fixation in 2.0% *v*/*v* glutaraldehyde in phosphate buffer (pH 7.4), the sperm pellet was washed for 15 min in fresh buffer containing 4% *w*/*v* sucrose, and embedded in 2% agar. Post-fixation was performed using 1% osmic acid in phosphate buffer. Samples embedded in agar were subsequently dehydrated in a graded series of ethanol. After dehydration, small pieces of agar containing spermatozoa were further embedded in Epon resin (Polysciences Inc., Warrington, PA, USA). Sections were cut on a Reichert OmU2 ultramicrotome (Reichert-Jung AG, Vienna, Austria) with a diamond knife. Ultrathin sections (70 nm) were collected on Parlodion 0.8%/isoamyl acetate-coated 100 mesh Nickel grids (EMS, Fort Washington, PA, USA) and counterstained with 2% uranyl acetate and lead citrate before observation. Sections were examined with a Zeiss transmission electron microscope 902 (Leo, Rueil-Malmaison, France). Images were acquired using a Gatan Orius SC1000 CCD camera (Gatan France, Grandchamp, France).

## Figures and Tables

**Table 1:** Detailed semen parameters for the three patients harboring *DNHD1* mutations.

	DNHD1__1_	DNHD1__2_	DNHD1__3_	Normal Range
Specimen characteristics				
Abstinence duration	nt	7	5	2–7
Volume (mL)	3.2	4	3.25	1.5–7
pH	8.1	7.9	7.9	7.2–8.0
Viscosity	normal	normal	normal	-
Numeration				
Sperm count (10^6^/^mL^)	0.3	40	1.54	15–200
Total numeration	0.97	160	4.65	>39
Round cells (10^6^/^mL^)	4	nt	2.25	<1
Polynuclear (10^6^/^mL^)	<1	nt	<1	<1
Motility				
Motile sperm (%)	20	0	10.5	>40
Progressive sperm (%)	10	0	3.5	>32
Non-progressive sperm (%)	10	0	6.5	>8
Immotile sperm (%)	60	100	89.5	<60
Other tests				
Viability (% alive)	nt	79	73	>58
Fructose, seminal (mg/dL)	nt	300	nt	120–450
Morphology				
Normal morphology (%)	9	0	4	>4
Abnormal morphology (%)	91	100	96	<96
*Multiple anomalies index*	1.51	nt	2.3	*-*
Head anomaly	32	10	96	-
*Elongated*	0	nt	0	*-*
*Thinned*	0	nt	8	*-*
*Microcephalic*	1	nt	12	*-*
*Macrocephalic*	1	nt	1	*-*
*Duplicated*	0	nt	6	*-*
*Abnormal base*	0	10	47	*-*
*Malformed acrosome*	30	nt	89	*-*
Intermediate piece anomaly	3	nt	15	-
*Cytoplasmic residue*	1	nt	1	*-*
*Thin*	0	nt	5	*-*
*Angulation*	2	nt	9	*-*
Flagellum anomaly	18	90	43	-
*Absent*	7	nt	0	*-*
*Short*	3	90	0	*-*
*Irregular size*	0	nt	2	*-*
*Coiled*	5	nt	39	*-*
*Multiple*	1	nt	2	*-*

nt, not tested.

## Data Availability

Not applicable.

## Supplementary Materials

The following are available online at https://www.mdpi.com/article/10.3390/ijms24032559/s1.
