# Supplementary material for: New Mutations in DNHD1 Cause Multiple Morphological Abnormalities of the Sperm Flagella
Source: Int J Mol Sci. 2023 Jan 29;24(3):2559. doi: 10.3390/ijms24032559 (PMC9916431; doi:10.3390/ijms24032559)
Supplement: Supplementary file 1 [file ijms-24-02559-s001.zip › Table S1.pdf]

Table S1. Detailed description of the variants in *DNHD1* identified by whole exome sequencing in the cohort of 167 MMAF individuals.

Patients	Nationality	Gene	Variant coordinates (hg38)	Transcript	cDNA Variation	Amino acid variation	Allelic status	Allele frequency (gnomAD v2.1)	Mutation Taster	PolyPhen-2	SIFT	CADD
DNHD1_1	European	*DNHD1*	chr11:6558077	NM_144666.3	c.8782C>T	p.(Arg2928Ter)	Hom	0.00114	DC	NA	NA	34
DNHD1_2	Iranian	*DNHD1*	chr11:6546928	NM_144666.3	c.5989G>A	p.(Gly1997Ser)	Hom	0.000192	DC	PD	D	26.1
DNHD1_3	European	*DNHD1*	chr11:6533756	NM_144666.3	c.2581C>T	p.(Arg861Cys)	Het	0.000367	DC	PD	D	24.4
		DNHD1	chr11:6546970	NM_144666.3	c.6031C>T	p.(Arg2011Trp)	Het	0.0000751	DC	LB	D	23

PolyPhen-2, Polymorphism Phenotyping v2; SIFT; Sorting Intolerant From Tolerant; CADD, Combined Annotation Dependent Depletion; gnomAD, Genome Aggregation Database; Hom, homozygous; Het, heterozygous; D, damaging; PD, probably damaging; DC, disease causing; LB, likely benign; NA, not available.
