# Supplementary material for: New Mutations in DNHD1 Cause Multiple Morphological Abnormalities of the Sperm Flagella
Source: Int J Mol Sci. 2023 Jan 29;24(3):2559. doi: 10.3390/ijms24032559 (PMC9916431; doi:10.3390/ijms24032559)
Supplement: Supplementary file 1 [file ijms-24-02559-s001.zip › Table S2.pdf]

Table S2. Primer sequences used for Sanger sequencing verification of *DNHD1* variations.

Name	Sequence (5'>3')	Tm	Length
**DNHD1_ex14-P0503-F**	TCTTACCCTGTCCATGTGGC	60	485
**DNHD1_ex14-P0503-R**	GCCTTCTCACCGAGATGTCC	60	
**DNHD1_ex21-P0503-F**	ACTGCGCTCACCACTGTTTA	60	485
**DNHD1_ex21-P0503-R**	GCCTCTTTTGGCCCATGTTG	60	
**DNHD1_ex21-P0233-F**	CTCTAGAGCGTGAGCTGGTG	59.9	670
**DNHD1_ex21-P0233-R**	CAGTGCTGGATCCCGATTGA	59.82	
**DNHD1_ex25-P0132-F**	TACCTGGAACGACAGTGGGA	60.18	500
**DNHD1_ex25-P0132-R**	CCTCACCTCTGCAACACCAT	59.96	

Tm: melting temperature
