# Supplementary figures and images for: New Mutations in DNHD1 Cause Multiple Morphological Abnormalities of the Sperm Flagella
Source: Int J Mol Sci. 2023 Jan 29;24(3):2559. doi: 10.3390/ijms24032559 (PMC9916431; doi:10.3390/ijms24032559)

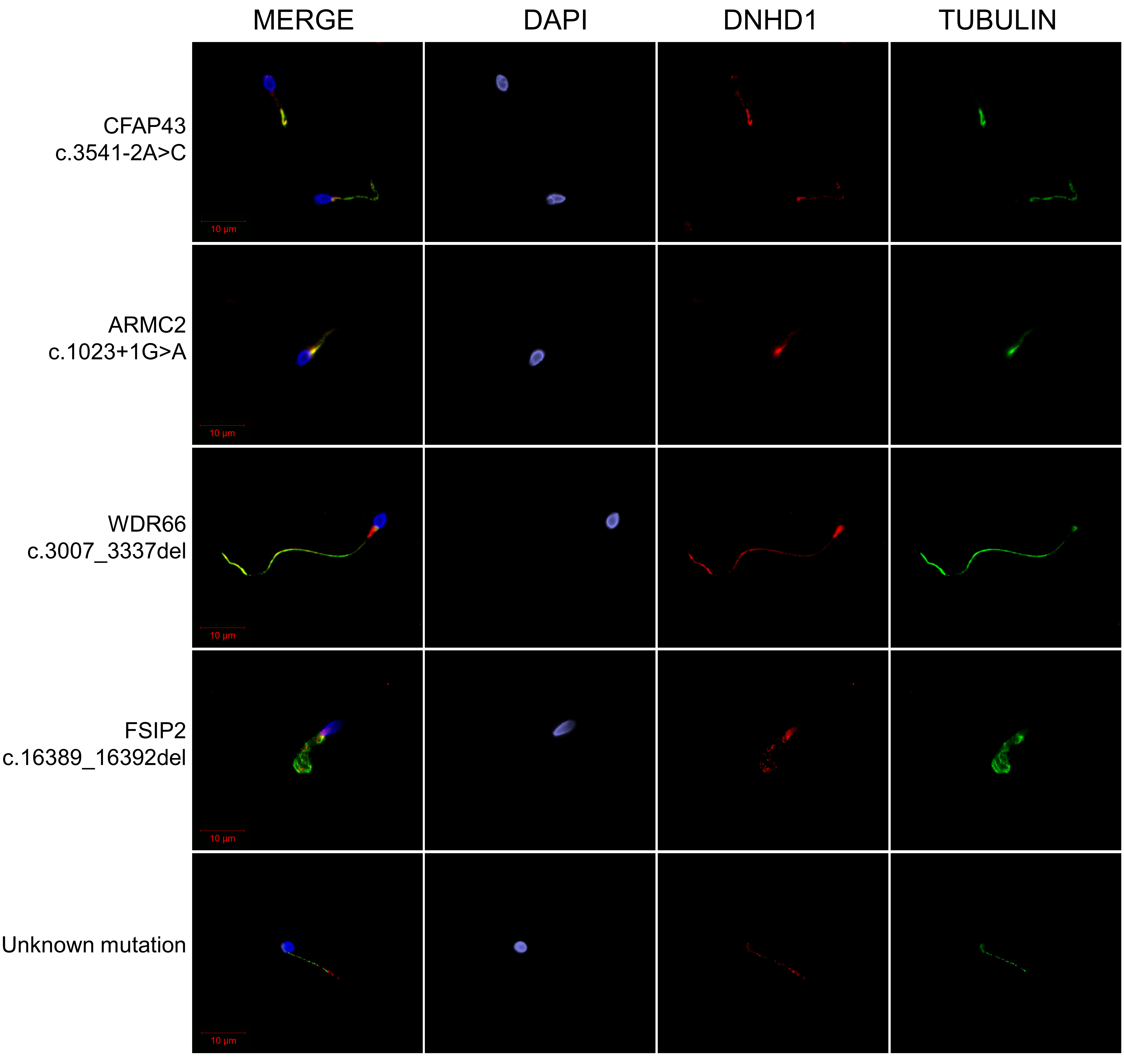

Supplement: Supplementary file 1 [file ijms-24-02559-s001.zip › Figure S1.tif]
